# Fracture Modeling of QP980 Steel: Evaluating the Rice–Tracey and DF2016 Criteria Under Diverse Loading States

**DOI:** 10.3390/ma18061303

**Published:** 2025-03-15

**Authors:** Hammad Akhtar, Thamer Sami Alhalaybeh, Xucheng Fang, Salah Ud Din Asbah, Shuaijun Chao, Yanshan Lou

**Affiliations:** School of Mechanical Engineering, Xi’an Jiao Tong University, 28 Xianning West Road, Xi’an 710049, China; ha.mes@stu.xjtu.edu.cn (H.A.); thamersami@stu.xjtu.edu.cn (T.S.A.); asbah@stu.xjtu.edu.cn (S.U.D.A.);

**Keywords:** ductile fracture, advanced high strength steel, strain path, sheet metal forming

## Abstract

The ductile fracture behavior of QP980 steel was studied under various loading conditions, including shear (SS), equibiaxial tension (EBT), plane strain tension (PST), and uniaxial tension (UT). The experimental results are compared to the predictions from the Rice–Tracey and DF2016 criteria. Excluding the Lode parameter restricts the Rice–Tracey criteria, which considers stress triaxiality only, from making reasonable predictions of fracture behavior under complicated loading conditions of PST and SS. However, it yields reasonable predictions for simple stress states, UT, and EBT. The DF2016 criteria take both the Lode parameter and stress triaxiality into account and lead to a reasonable prediction over the maximum range of stress states. Experimental findings verify that the DF2016 model adequately describes the fracture initiation and propagation mode under conditions of moderate and high triaxiality. The findings show that the DF2016 criterion presents a more robust and versatile approach toward modeling ductile fracture behavior in QP980 steel for applications in structural engineering and the automobile industry, where accurate failure modeling is important.

## 1. Introduction

As industrial civilization has grown in recent years, it has become increasingly concerned with environmental preservation, energy efficiency, and safety in the transportation process. Reducing the weight of materials is one way to address the aforementioned issues. Due to its great strength, AHSS (Advanced High Strength Steel) is often used in the construction of body reinforcements, such as crash beams, A/B beam-columns, and door frame reinforcements [1]. The use of AHSS may decrease plate thickness and improve crash performance, which lowers the car’s overall quality and lowers energy and fuel consumption [2]. However, it is easily broken in crashes due to its weak ductility, which has become a significant safety concern. To improve the security and application efficiency of QP980 steel plate, experiments must be designed to observe its plastic behavior under complex load conditions [3]. Then, using numerical simulation, the corresponding yield function and fracture criterion must be calibrated to achieve an accurate prediction of load capacity and fracture stroke.

To statistically describe the yield behavior of metals, researchers have created a variety of yield criteria. First, by taking into account the influence of pressure and the third stress invariant, several isotropic yield functions were created to increase the modeling accuracy of yielding at various stress states, such as compression, shear, tension, etc. Tresca, von Mises, Drucker, Drucker–Prager, and others are examples of these yield functions. When the sheet metals are rolled, texture is created, and their plastic behavior is dependent on the direction of loading. Numerous anisotropic yield functions were therefore put forward. One of the most representative research findings is the Hill48 yield criterion [4,5]. The plane stress non-quadratic yield criteria were proposed by Barlat et al. [6] to characterize an-isotropic metal sheets, including sheets of aluminum alloy. Based on a similar approach, Barlat et al. [7] created more precise anisotropic yield functions to better describe the anisotropic behavior of metals and alloys. Banabic et al. [8], Aretz and Barlat [9], Cazacu et al. [10], Yoshida et al. [11], Lou and Yoon [12], and others have presented other well-known anisotropic yield functions. Over the last 15 years, a number of studies have examined anisotropic hardening, including those by Stoughton and Yoon [13], Lee et al. [14], Park et al. [15], Hou et al. [16,17,18], Hu et al. [19,20,21,22,23], and Du et al. [24]. Recently, Hu et al., Lou et al. [25,26,27], and others have modeled plastic behavior under different stress situations. These developments significantly increase the accuracy of simulating metals’ plasticity under a variety of loading scenarios and stress levels.

Since the 15 fracture tests of AA2024-T351 by Bao and Wierzbicki, ductile fracture has been the subject of increased research during the last 20 years. The modified Mohr–Coulomb criterion [28], the DF2012 [29], the DF2016 [30,31], Mu [32], Ganjiani-Homayounfard [33], Hu-Chen [34], Zheng [35], Zhang [36], Quach [37], Shang et al. [38], and others were among the several ductile fracture criteria that were subsequently created. These criteria, which are based on the nucleation, development, and coalescence of voids, —micro processes of ductile fractures—are given in the form of mixed stress and strain [39]. Lou and Stoughton [40,41], Khan [42], and others presented the stress-based ductile fracture criteria. In the last ten years, an anisotropic ductile fracture has also been investigated. Jia and Bai [43], Luo et al. [44], and Beese et al. [45] have presented modeling techniques for anisotropic ductile fracture. Lou et al. [46] assessed the fracture loci derived from fracture criteria by comparing them to experimental results for Al2024-T351. Their comparison showed that the modified Mohr–Coulomb criterion, along with a newly proposed criterion, offers adequate predictability of fracture strain. Ductile fracture modeling in pre-cracked tensile testing of SUS304L stainless steel was investigated numerically by Park et al. [47]. The ductile fracture and plasticity of an Al-Si-Mg die-cast alloy was modeled by Baral et al. [48]. Based on the micro processes of ductile fracture—strain controlled void nucleation, triaxiality-governed void development, and shear coalescence of voids—Luo et al. [49] presented the ductile fracture criteria. By considering two main void deformation modes, Mu et al. [50] created a mathematical model of ductile fracture behavior. They then used a hybrid experimental–numerical approach to calibrate the model for DP780. In order to correctly define the deformation behavior of sheet metal under complicated stress states, the aforementioned study aims to examine the ductile fracture behavior of sheet metals under various stress triaxialities. Conversely, the ductile fracture behavior of sheet metals is mostly simulated using a single ductile fracture criterion. Consequently, the ductile fracture behavior of QP980 under complicated stress conditions was characterized using a variety of ductile fracture criteria. Recent studies have found that the uncoupled models are as accurate in predicting the ductile fracture behavior as the coupled models, despite the fact that they have fewer model parameters [51,52]. In this regard, the model could be suitable for use in civil and automotive engineering applications that need extensive structural modeling. Equivalent fracture strain is used to create the fracture locus in three dimensions [53,54].

The fracture characteristics of QP980 steel under uniaxial stress, plane strain tension, shear, and equibiaxial tension aligned with the rolling direction (RD) are examined, incorporating both modeling and experimental results. Two common ductile fracture criteria are converted into a generic 3D stress space, and their fracture loci are shown in the space of (η,L,C). The material’s fracture behavior is predicted and assessed using the DF2016 and Rice–Tracey criteria. Particular emphasis is placed on the relationship between the equivalent plastic strain to fracture and stress triaxiality, as well as the Lode parameter. Furthermore, the cut-off value for stress triaxiality is determined and compared across each ductile fracture criterion.

## 2. Experiments

**Figure 1:**
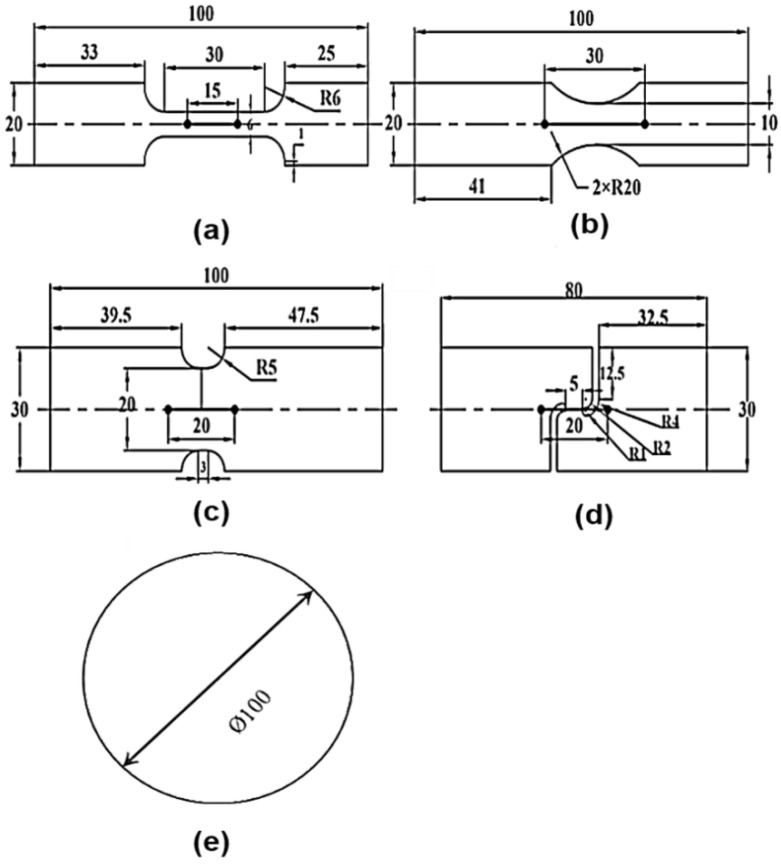
Five designs of specimen: (**a**) dog-bone specimen; (**b**) notched specimen with a radius of 5 mm; (**c**) notched specimen with a radius of 20 mm; (**d**) in-plane shear specimen; (**e**) bulging specimen; [unit: mm].

**Figure 2:**
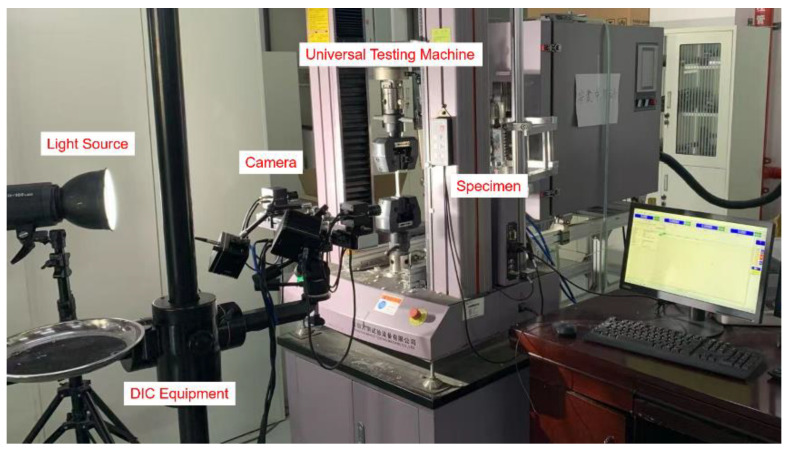
Universal testing machine and the XTOP digital image correlation system.

A laser cutting machine is used to cut all the specimens from QP980 sheet metal in the direction of RD, where UT, PST, SS and EBT geometry are shown in Figure 1. Universal testing machine is utilized to perform tensile tests on dogbone specimens, nothchR5, nothchR20, and shear samples, as shown in Figure 2. The machine is configured to operate at a speed of 1.8 mm/min for dogbone specimens and 0.5 mm/min for nothchR5, nothchR20, and shear. Table 1 presents the chemical composition of QP980 steel, with a thickness of 1.0 mm, represented in weight percentage (wt%).

The plastic behavior of materials subject to uniaxial tension is exemplified by the dog-bone specimen. Fracture behavior under the plane strain tension is represented by the notched specimen, while the shear fracture is characterized by the in-plane shear specimen. For each test, a total of approximately 200 to 250 images are captured utilizing the digital image correlation system (DIC) at the macroscopic level. The specimens were coated with white paint, which is marked with small black dots prior to testing. The areas exhibiting uniform deformation were chosen for the placement of the extensometer to ensure accurate measurement results, as the deformation at both ends of the parallel region in the center of the specimen may not consistently be uniform. Due to the non-uniform deformation observed at both ends of the parallel area in the center of the specimen, the region exhibiting uniform deformation is selected for the placement of the extensometer. This approach ensures the acquisition of precise measurement data. The test result for QP980 is obtained under equibiaxial stress conditions utilizing a bulging test, with a blank holding force set at 600 kN. To guarantee adequate reproducibility and reliability of the specimens, the rolling loading direction is evaluated by a minimum of three tests.

**Figure 3:**
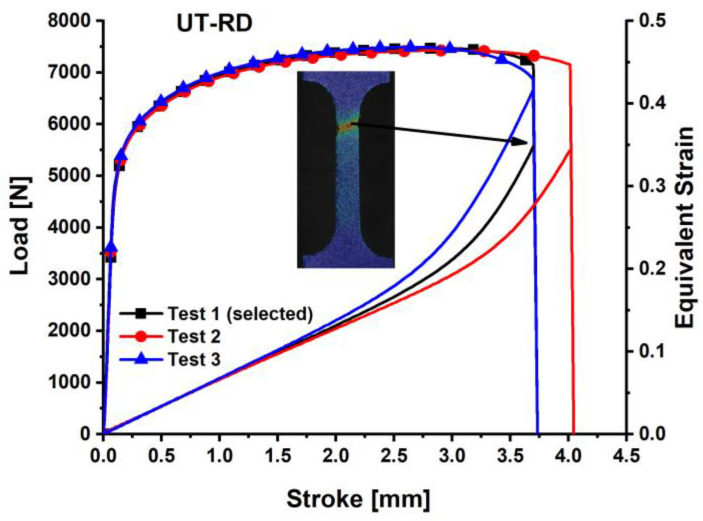
Load–stroke curves of QP980 for the dogbone specimens (UT) along RD.

**Figure 4:**
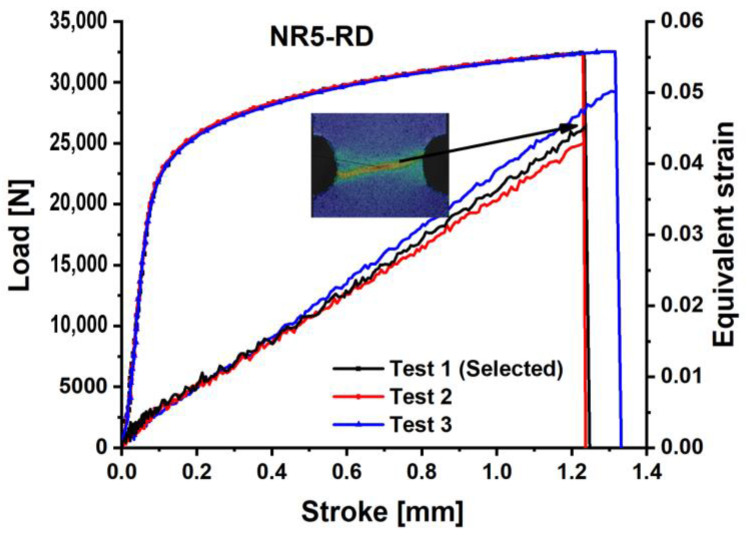
Load–stroke curves of QP980 for the Notched R5 specimens (NR-5) along RD.

**Figure 5:**
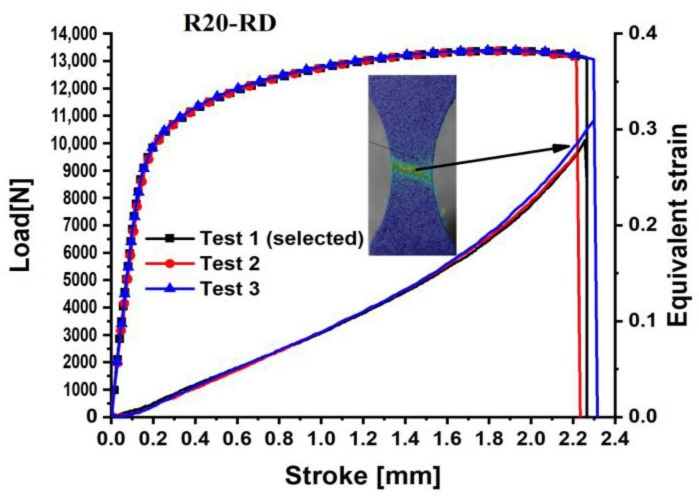
Load–stroke curves of QP980 for the Notched R20 specimens along RD.

**Figure 6:**
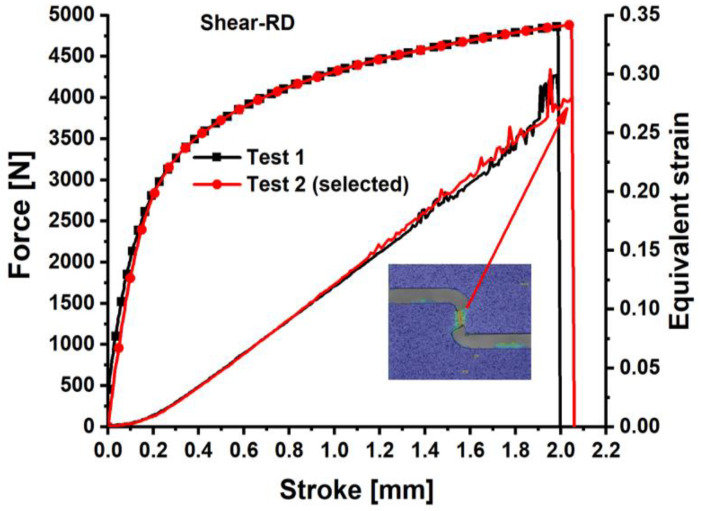
Load–stroke curves of QP980 for the shear specimens along RD.

The load–stroke curves for the dogbone specimens are shown in Figure 3, the notched specimens with a 5 mm radius are depicted in Figure 4, for the notched specimens with a 20 mm radius are shown in Figure 5, and for the shear specimens are represented in Figure 6. The reproducibility of the tests concerning the material’s hardening action is reliable. Nonetheless, especially for the shear test, the hardening tendency exhibited more reproducibility than the failure stroke. Poor reproducibility in the failure stroke may result from an inhomogeneous microstructure, manufacturing errors, or similar factors. The most repeatable tests with the mean stroke at failure were selected to represent the experimental results for the varied specimens. Consequently, test #1 is selected for the subsequent analysis of the dogbone, notched R5, and R20 specimens, while test #2 is designated for the shear specimen.

**Figure 7:**
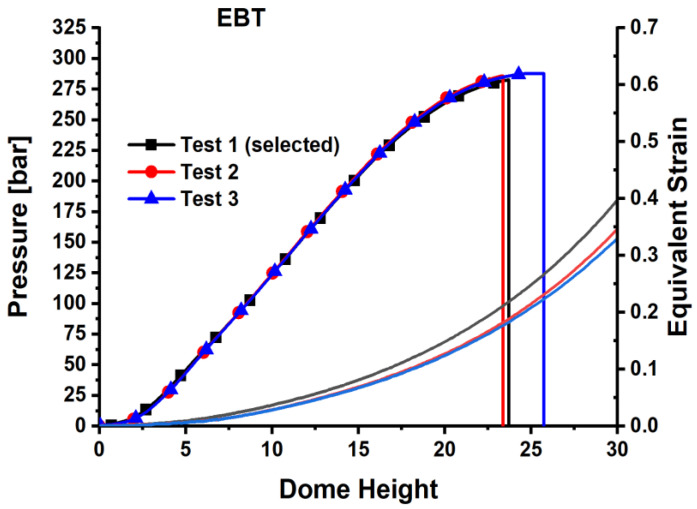
Bulging experimental results of QP980.

**Figure 8:**
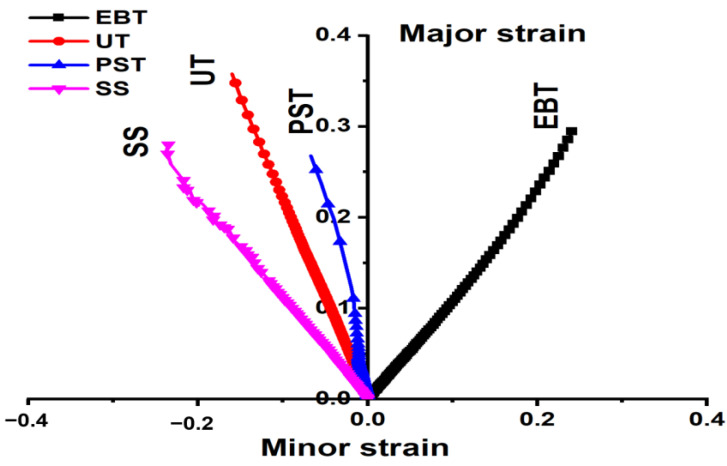
Strain paths in major–minor strain space for four tests up to fracture.

Bulging tests were conducted on QP980 steel. The velocity of the punch was 5 mm/min. To ensure the experiments are replicable, three bulging tests were conducted. Figure 7 illustrates a graph showing the pressure and dome height. The graph illustrates the progression of equivalent strain in proportion to the dome height, with test #1 selected for further analysis. Figure 8 illustrates compressive fracture for strain routes in major–minor strain space for UT, PST, SS, and EBT tests up to the point of fracture. The graphs illustrate the strain limitations prior to fracture, with the X-axis representing minor strain and the Y-axis indicating major strain. The area under the fracture represents the secure formation zone, whereas locations on or above them indicate failure. This graphic enhances forming techniques, guaranteeing that QP980 steel satisfies automotive performance standards.

## 3. Ductile Fracture Criteria

The main processes that control the ductile fracture in QP980 consist of void nucleation, growth, and coalescence, which are affected by loading conditions, strain rate, and microstructure. Establishing a reliable ductile fracture criterion is crucial for predicting the material’s behavior under various loading conditions for UT, PST, SS, and EBT. This investigation explores QP980 steel under different stress conditions, clarifying the fracture criterion linked to stress triaxiality, the Lode parameter, and equivalent plastic strain to improve understanding of the material’s fracture behavior. Table 2 presents a comparison of the Lode, fracture strain, and triaxiality under RD for QP980 steel, as obtained from the strain path analysis. The findings illustrate the impact of different specimens EBT, PST, UT, and SS, on the fracture characteristics of the material QP980.

### 3.1. DF2016 Fracture Criterion

There have been reports of the recent development of a micro-mechanism-motivated phenomenological ductile fracture model. Based on the observation of certain micro-mechanisms, it has been determined that the process of ductile fracture is the nucleation, growth, and coalescence of voids. The DF2016 fracture criterion is widely used to predict the fracture strain of metal because of its high accuracy for a variety of loading conditions.

(1) $${\left(\frac{2\tau_{max}}{{\bar{\sigma }}_{VM}}\right)}^{C_{1}}{\left(\langle \frac{f\left(\eta ,L,C\right)}{f\left(1/3,-1,C\right)}\rangle \right)}^{C_{2}}\varepsilon_{f}=C_{3}\;\langle x\rangle =\left\{\begin{array}{cc} x & if\;x\geq 0\\ 0 & if\;x<0 \end{array}\right..$$

To describe the impact of the Lode parameter on the cut-off value, Lou et al. [55] added the material parameter $C_{4}$ to Equation (4), which implies that the function *f*(*η*, *L*, *C*) is envisaged to be written in the form of

(2) $$\;{f\left(\eta ,L,C\right)=\eta +C}_{4}\frac{\left(3-L\right)}{3\sqrt{L^{2}+3}}+C.$$

In this context, *τ_max_* represents the peak shear stress, while $C_{1}$, $C_{2}$, $C_{3}$, and $C_{4}$ denote four distinct fracture parameters. Additionally, C is incorporated to account for the influence of *L* on the alteration of void shape throughout the deformation process. The influence of *τ_max_* on ductile fracture can be likened to that of *L* on the coalescence of shear voids. Equation (1) may also be represented as

(3) $${\left(\frac{2}{\sqrt{L^{2}+3}}\right)}^{C_{1}}{\left(\left\langle \frac{f\left(\eta ,L,C\right)}{f(\frac{1}{3},-1,C}\right\rangle \right)}^{C_{2}}\varepsilon_{f}=C_{3}.$$

In general, the ductile fracture is regarded as a continuous process in the numerical prediction sense. Therefore, Equation (1) can be expressed in an integral form as follows:

(4) $$D=\int_{0}^{\varepsilon_{f}}\frac{1}{C_{3}}{\left(\frac{2}{\sqrt{L^{2}+3}}\right)}^{C_{1}}{\left(\frac{f\left(\eta ,L,C\right)}{f\left(\frac{1}{3},-1,C\right)}\right)}^{C_{2}}d\varepsilon^{p},$$

where *D* is the value of cumulative damage, which changes from 0 to 1. Failure occurs for materials when *D* = 1. $C_{1}$ governs the nucleation of voids, $C_{2}$ pertains to the growth of voids, $C_{3}$ is associated with the equivalent strain leading to fracture, and $C_{4}$ characterizes the impact of Lode parameter on the fracture behavior.

By fitting the testing data from Table 2, the fracture constants ($C_{1\;}$= 0.7299, $C_{2\;}$= 0.9895, $C_{3\;}$= 0.2295, and $C_{4\;}$= −0.2948) are determined. The parametric study provides a comprehensive analysis of how the model coefficients influence fracture prediction. The investigation focuses solely on the effects of parameters $C_{1}$, $C_{2}$, $C_{3}$, and $C_{4\;}$on the fracture locus established within the principal strain space for the purpose of simplification. Parameters $C_{1\;}$and $C_{2\;}$adjust the ratio of the fracture strain under uniaxial tension to that under other stress states. The influence of the shear mechanism on fracture behavior increases progressively with parameter $C_{1}$, whereas the fracture strain decreases. The asymmetry of the fracture locus in relation to *L* = 0 diminishes, as the parameter $C_{2\;}$increases. Furthermore, an increase in parameter $C_{2\;}$may result in the fracture behavior not occurring under certain loading conditions. Parameter $C_{3\;}$exclusively modifies the magnitude of fracture strain without affecting its shape, while parameter $C_{4\;}$dictates the conditions under which the fracture occurs. The observed phenomena suggest that the DF2016 fracture criterion exhibits significant flexibility in characterizing fracture behavior as a result of the multi-parameter effect.

**Figure 9:**
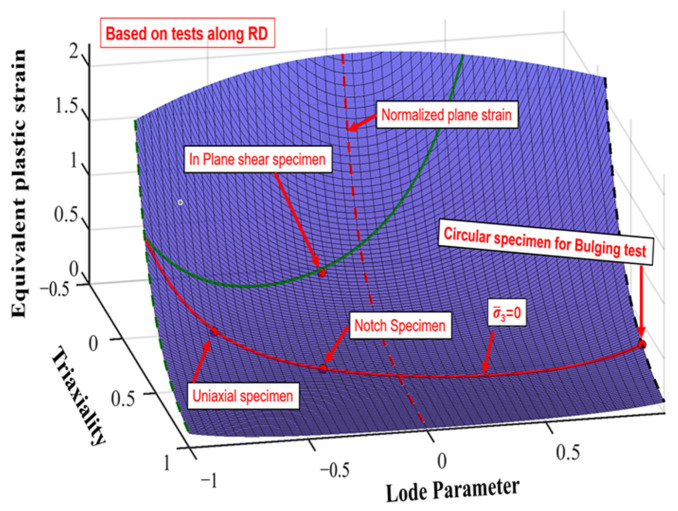
Fracture locus of QP980 constructed by the DF2016 criterion along RD.

The computed load–stroke curves for the DF2016 fracture criteria closely resemble the real curves across several stress levels, exhibiting little deviation. Figure 9 shows the fracture locus of QP980 steel based on the DF2016 criterion in terms of triaxiality, the Lode parameter, and equivalent plastic strain to fracture. Experimental test points from various specimens, including uniaxial tension, plane strain tension, shear, and equibiaxial tension, confirm the predictive capability of the DF2016 criterion over a wide range of stress states. The model incorporates triaxiality and the Lode parameter in an effective manner, which makes it a powerful tool for the analysis of ductile fracture behavior.

### 3.2. The Rice–Tracey Criterion

Rice and Tracey [56] have investigated the development rate of a singular spherical cavity inside an infinite isotropic elastic material, subjected to minor deformations under external normal loads. Certain problems of non-uniform stress and related stress state expressions indicate that void expansion across various stress levels may be adequately described by a semi-empirical equation:

(5) $$\;\int_{0}^{{\bar{\varepsilon }}_{f}}0.283exp\left(\frac{3}{2}\eta \right)d\bar{\varepsilon }=C_{5}.$$

The Rice–Tracey criteria posited that the equivalent plastic strain at fracture is only contingent upon stress triaxiality and insensible to the Lode parameter.

**Figure 10:**
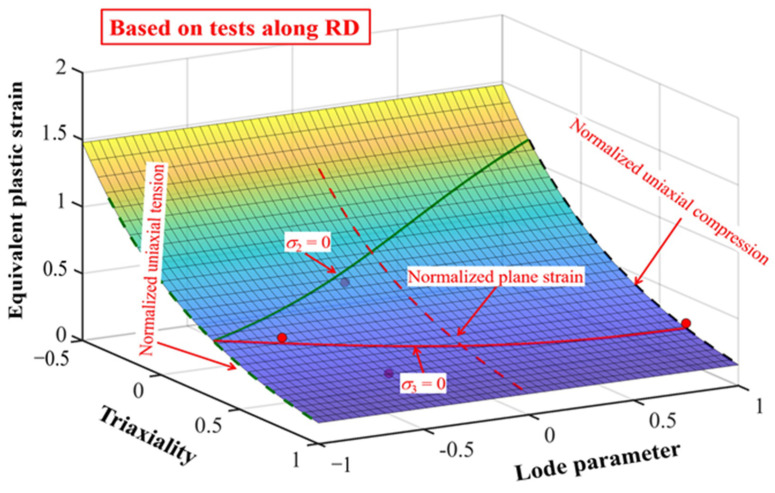
Fracture locus of QP980 constructed by the Rice–Tracey criterion along RD.

In the Rice–Tracey criteria, the fracture constant (*C*_5_ = 0.1976) assessed from the experimental data points of QP980 steel shown in Table 2 to achieve the minimal error function which equals 1.1510. A comparison between the experimental data points and the fracture location determined using the Rice–Tracey criterion is shown in Figure 10. Increased stress triaxiality lowers the equivalent plastic strain at fracture, and the Lode parameter has no effect on the Rice–Tracey fracture locus configuration. Additionally, as there is no cut-off plane for stress triaxiality within the Rice–Tracey fracture locus, all three branches of plane stress are represented on the fracture locus generated from the Rice–Tracey criteria.

A comparative study of the DF2016 criteria with the Rice–Tracey model was conducted for the predictability of the fracture behavior of the ductile fracture of the QP980 steel under varying conditions of the load. To predict the fracture behavior of steel, Rice–Tracey model is widely used under various stress states. The triaxiality of the stress is the most dominant factor for the fracture that the Rice–Tracey model identifies, and it is appropriate for the conditions of the uniaxial and the conditions of the equibiaxial tension loads. In contrast, the DF2016 criterion expands the applicability of the Rice–Tracey approach by including the effects of both stress triaxiality and the Lode parameter, providing a more complete framework for the prediction of fracture initiation. The limitation of traditional models and the potential benefits of a modern formulation was compared, which identified the most appropriate criterion for QP980 material under various loading conditions.

The calibration of the fracture parameters for the two-component DF2016 criterion was conducted using the unconstrained optimization method in MATLAB R2024a, based on the data in Table 2. The error function is defined as follows:

(6) $$error=\sum_{1}^{n}{\left(\frac{\varepsilon_{f}^{exp}-\varepsilon_{f}^{pre}}{\varepsilon_{f}^{pre}}\right)}^{2},$$

where *n* represents the total amount of fracture data, which is equal to 4 in this section, and $\varepsilon_{f}^{exp}$ and $\varepsilon_{f}^{pre}\;$signify the experimental and predicted fracture strains, respectively.

**Figure 11:**
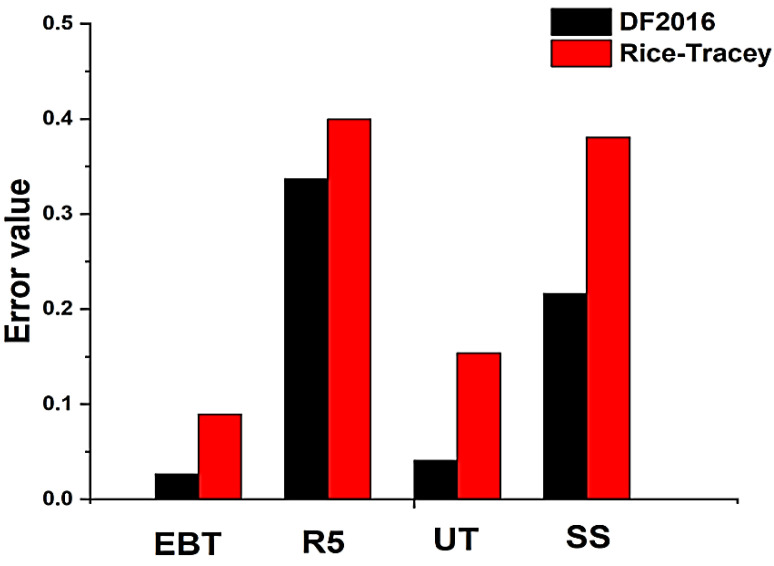
Value of errors under the Rice–Tracey and DF2016 models at various loading conditions.

The error values for the DF2016 model and the Rice–Tracey model under different loading conditions are shown in Figure 11, which indicate that the DF2016 model is more accurate. In the SS test, the Rice–Tracey model shows the largest error, highlighting its limitations in shear-dominated conditions due to its complete reliance on triaxiality. In contrast, DF2016, which includes the load parameters, clearly reduces the prediction errors in all loading conditions. It shows that it is more flexible and accurate at predicting when a fracture initiates.

## 4. Results and Discussion

**Figure 12:**
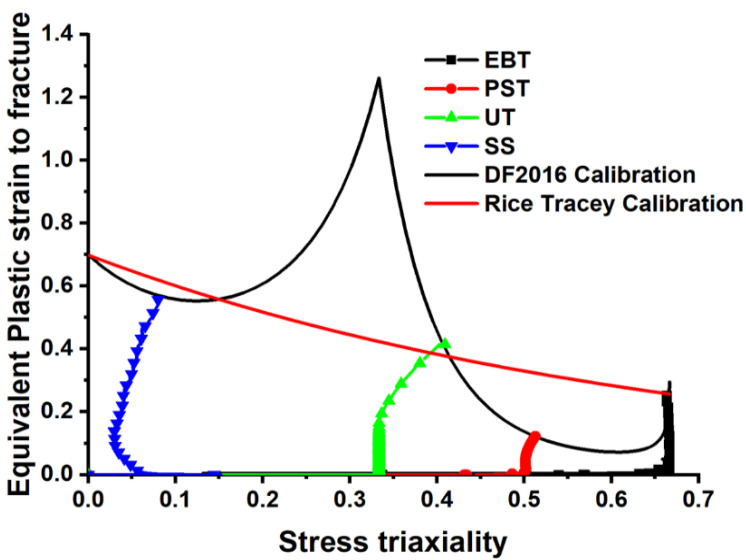
Comparison of experimental fracture data for QP980 steel with the DF2016 and Rice–Tracey criteria.

The experimental results are compared to the predictions by using the Rice–Tracey and the DF2016 fracture criteria for analyzing the ductile fracture behavior and the fracture of QP980 steel. Based on the Rice–Tracey criteria, the stress triaxiality is the only factor that impacts the ductile fracture, and it predicts that the fracture strain declines exponentially with the increasing triaxiality. As shown in Figure 12, uniaxial tension and equibiaxial are two cases of fundamental stress states for which the Rice–Tracey criteria effectively fit the experimental fracture data. However, it excludes the Lode parameter, a vital factor to affect fracture behavior in more complicated loading conditions, such as shear and plane strain tension. Consequently, obvious discrepancies between the experiment and a model prediction are observed in the stress states, where triaxiality is apparent and the strain route is complex. The DF2016 criteria significantly enhance the model’s prediction capability under UT, PST, SS, and EBT. They enable the model to correctly capture how these materials fail under complex states of stress. The model correctly identifies the trends of experimental data, particularly at intermediate and high triaxialities, where the Rice–Tracey model shows deviations and the DF2016 criteria may turn out to be very promising in predicting material failure for a broader range of stress conditions. Its reliability in simulating the onset and propagation of fractures is demonstrated by the good agreement of the fracture locations it predicts with experimental results.

Comparison of the two models shows that the DF2016 criterion is superior to the Rice–Tracey criterion. Although it is restricted from application in more complex loading situations as it ignores the Lode parameter, the Rice–Tracey model can work effectively for simple stress states. The fracture behavior of QP980 steel can be described more thoroughly by the DF2016 criteria, which take into account the stress triaxiality and the load parameter. Experimental results confirm the superiority of the DF2016 model in predicting fracture initiation under different stress conditions.

The results demonstrate that stress triaxiality has a strong influence on fracture behavior. The Rice–Tracey criteria make good predictions in simple stress states, as they depend only on stress triaxiality. The Lode parameter, introduced into the DF2016 criteria, leads to higher accuracy for predicting fracture initiation under complex stress states. This investigation shows that the DF2016 criteria give a better method of studying the fracture behavior of QP980 steel.

## 5. Conclusions

This study evaluates the fracture behavior of QP980 steel under various stress states and compares the predictions based on the Rice–Tracey and DF2016 criteria. The Rice–Tracey criterion is satisfactory for simple stress states; however, it is unsatisfactory under complex conditions, as it neglects the Lode parameter. The DF2016 criterion, which incorporates stress triaxiality and the Lode parameter, shows higher accuracy in terms of predicting the fracture behavior under simple and complex states. The DF2016 model has been proven to be a robust tool for analyzing and predicting the ductile fracture of QP980 steel; therefore, it increases the potential for such material in many applications requiring good modeling of materials failure under multiple loading conditions.

## Figures and Tables

**Table 1:** Chemical compositions of QP980 alloy (wt%).

C	Si	P	Mn	S	Al	Ti	N
0.18%	1.72%	0.01%	2.23%	0.002%	0.028%	≤0.1%	0.0033%

**Table 2:** Comparison of Lode, fracture strain, and traxiality under rolling direction.

Specimens	Lode Parameter L	$\mathrm{Fracture}\;\mathrm{Strain}\;{\bar{\varepsilon }}_{f}$	Stress Traxility η
EBT	0.99993	0.29473	0.64685
PST	−0.29995	0.12213	0.51275
UT	−0.7305	0.43002	0.4091
SS	−0.18434	0.55641	0.08003

## Data Availability

The original contributions presented in this study are included in the article. Further inquiries can be directed to the corresponding authors.
